# Epidemiology of classic Kaposi's sarcoma in the Israeli Jewish population between 1960 and 1998

**DOI:** 10.1038/sj.bjc.6601313

**Published:** 2003-10-28

**Authors:** E Guttman-Yassky, M Bar-Chana, A Yukelson, S Linn, R Friedman-Birnbaum, R Bergman, R Sarid, M Silbermann

**Affiliations:** 1 Department of Dermatology, Rambam Medical Center, Haifa, Israel; 2 Department of Anatomy and Cell Biology, The Bruce Rappaport Faculty of Medicine, Technion Israel Institute of Technology, Haifa, Israel; 3 Israel Cancer Registry, Israel Ministry of Health, Jerusalem, Israel; 4 Unit of Epidemiology, Rambam Medical Center, The Bruce Rappaport Faculty of Medicine, Technion Israel Institute of Technology, Haifa, Israel; 5 Phototherapy Unit, Department of Dermatology, Rambam Medical Center, Haifa, Israel; 6 The Bruce Rappaport Faculty of Medicine, Technion-Israel Institute of Technology, Haifa, Israel; 7 Faculty of Life Sciences, Bar Ilan University, Ramat Gan, Israel

**Keywords:** classic Kaposi's sarcoma, KSHV, Israeli Jews

## Abstract

Trends in the incidence of classic Kaposi's sarcoma in the Jewish population in Israel for the period between 1960 and 1998 were analysed. World standardised incidence rates of 20.7 and 7.5 per million among men and women, respectively, were calculated. The highest incidence rates were displayed by men originated from Africa and by Asian-born women.

Kaposi's sarcoma (KS) is a multifocal vascular tumour that predominantly presents as a multipigmented sarcoma of the skin. Classic Kaposi's sarcoma (CKS) is known as a clinicoepidemiological indolent variant of the disease primarily affecting the elderly (Sarid *et al*, 1999; Antman and Chang, 2000). Kaposi's sarcoma-associated herpesvirus (KSHV), also known as human herpesvirus 8 (HHV-8), is believed to be a major causative factor for all clinical variants of KS (Chang *et al*, 1994; Ablashi *et al*, 2002). In general, the seroprevalence rates of KSHV in different geographical regions correlate with the incidence rates of CKS (Gao *et al*, 1996; Chatlynne and Ablashi, 1999; Schulz, 1999; Antman and Chang, 2000). Yet, genetic and/or environmental cofactors affecting the risk of CKS after KSHV infection probably play an important role, and thus may modify the relationship between the seroprevalence of KSHV and incidence of KS (Ariyoshi *et al*, 1998; Goedert *et al*, 2002; Grossman *et al*, 2002).

The objective of the present study was to investigate the trends in the incidence of CKS in Israeli Jews between 1960 and 1998. The analysis of the trends of CKS in Jews living in Israel is unique, Israel being one of the countries with the highest incidence of CKS in the world, and a migration center for Jews from various countries of origin.

## METHODS

All KS cases reported to the population-based Israel Cancer Registry (ICR) between 1960 and 1998 were identified under skin cancer and sarcoma codes according to the International Classification of Diseases for Oncology Version 2 (ICD-O-2) morphology code 9140 (World Health Organization, 1979). ICR covers the entire Israeli population, and data quality is estimated to be high (Steinitz, 1989; Parkin *et al*, 1997). All recorded diagnoses were manually checked for registration accuracy; no misclassifications were found. No cases were reported on post mortem. KS cases that were diagnosed among immigrants before their arrival to Israel, among the Arab population, and in nonresidents were excluded. To identify the AIDS-associated KS, the ICR file was linked with the national HIV-seropositive and AIDS registries; 60 HIV-seropositive KS cases were excluded. The registry of HIV and AIDS cases in Israel is incomplete, due to the mandatory identification requirement. Yet, we believe that the majority of AIDS-KS cases are diagnosed and reported following their application for medical care.

ICR is linked to the Israel Population Registry, and receives information of date and country of birth, gender, immigration and mortality. Therefore, data retrieved from the ICR included these records, date of CKS diagnosis and topographical ICD-O codes. Using population data from the Population Registry at the National Bureau of Statistics (Central Bureau of Statistics, 1960–1998), age-, sex- and country of origin-specific incidence rates were calculated, as were age-standardised incidence rates, using the world standard population as a reference. Linear regression multivariate analysis was used to model incidence rates with respect to calendar period, age at diagnosis, gender and country or continent of origin (Breslow and Day, 1987).

## RESULTS

A total of 2107 cases of CKS (1475 men, 632 women, M/F=2.33) were registered in the entire Israeli Jewish population, during the 39-year study period (1960–1998).

Overall, the age-standardised incidence rates of CKS (±s.d.) in the Jewish population were 20.7 (±9.3) per million among men and 7.5 (±3.4) per million in women. The gender incidence ratio was 2.76 (RR=2.76 (20.7/7.5); 95% CI 1.57–3.95) and was rather constant during the study period (range 2.3–3.4). The total number of CKS cases for both genders combined for consecutive 10-year periods of diagnosis was 81 (3.8% of total cases) between 1960 and 1969, steeply increased to 396 cases between 1970 and 1979 (18.8%), followed by 574 cases (27.2%) between 1980 and 1989. A second substantial increase was observed during the period between 1990 and 1998, with 1056 new cases (50.1%). No significant trend in incidence was observed during other periods (Table 1

**Table 1:** Number of cases and age-standardised rates of classic Kaposi's sarcoma in Israeli Jews between 1960 and 1998

	**Men**			**Women**		
**Period**	**No. of cases**	**ASR**	**Crude rate**	**No. of cases**	**ASR**	**Crude rate**
1960–1964	23	5.1	4.47	8	2.2	1.59
1965–1969	39	6.5	6.54	11	1.9	1.88
1970–1974	117	16.7	17.31	44	6.0	6.54
1975–1979	163	19.3	21.41	72	7.8	9.42
1980–1984	171	17.6	20.05	74	6.7	8.8
1985–1989	236	21.6	26.44	93	6.8	10.31
1990–1994	384	30.4	37.45	161	9.9	15.38
1995–1998	342	29.7	37.61	169	10.7	17.99
1960–1998	1475	20.7	23.76	632	7.5	10.11

ASR: age-standardised rate (per 1 000 000).

).

A significant trend of increase in the mean age at diagnosis, in both genders, was evident. This might be due to the extended lifespan. In men, the mean age (±s.d.) increased from 64.9 (±13.2) to 69.5 (±13.9) during the period between 1960 and 1998 (*P*<0.01). In women, there was an even more pronounced increase in the mean age at diagnosis (±s.d.), from 51.9 (±18.1) to 73.5 (±12.7) (*P*<0.01). The age-specific rates increased from 10.2 per million in men and 2.5 per million in women in the age group of 35–44, to 300.8 per million in men and 119.2 per million in women in the age group of 75 or more. Among the age group of 0–24, the rates per million were 0.6 and 0.2 in males and females, respectively, with no cases recorded below 5 years of age (Table 2

**Table 2:** Age-standardised incidence rates (based on world standard population) of classic Kaposi's sarcoma among Israeli Jews by gender and patient age group between 1960 and 1998

	**Men**		**Women**	
**Age group (years)**	**No. of cases**	**Rate per million**	**No. of cases**	**Rate per million**
0–24	17	0.6	6	0.2
25–34	45	5.1	17	1.9
35–44	75	10.2	19	2.5
45–54	110	18.9	32	5.2
55–64	261	53.9	85	16.3
65–74	417	121.4	203	51.8
⩾75	550	300.8	270	119.2
Total	1475	20.7^a^	632	7.5^a^

a Age-standardised rate (per 1 000 000).

). The substantial increase with age in the age-specific rates of CKS cases is consistent with other reports (Cottoni *et al*, 1996; Hjalgrim *et al*, 1996; Brenner *et al*, 2002).

Immigrants born in Asia, Africa, Europe and America exhibited a strong increase in incidence rates during the late 1960s and the early 1970s, and during the early 1990s. Israel-born Jews demonstrated a similar increase in incidence during the late 1960s and the early 1970s but failed to show the second strong increase during the 1990s.

Analysis of CKS incidence rates by continent of origin revealed highly significant (*P*<0.01) differences in incidence rates (Table 3

**Table 3:** Age-standardised incidence rates of CKS among Israeli Jews by geographical origin, calendar period of diagnosis and gender

	**Israeli-born**		**Asian-born**		**African-born**		**European- and American-born**	
	**Men**	**Women**	**Men**	**Women**	**Men**	**Women**	**Men**	**Women**
*1960–1964*								
No.	2	1	2	2	5	0	13	5
ASR	7.3	5.6	2.3	1.9	11.4	0	4.5	1.9

*1965–1969*								
No.	1	1	3	0	7	1	28	9
ASR	4.5	0.2	2.8	0	11.2	0.8	6.9	2.3

*1970–1974*								
No.	14	5	20	7	15	4	67	28
ASR	32.8	7.2	16.6	5.8	20.6	4.8	18.6	6.4

*1975–1979*								
No.	12	6	26	13	35	14	90	39
ASR	20.3	6.7	17.6	7.8	36.5	13.3	16.8	6.2

*1980–1984*								
No.	16	3	30	8	27	15	97	47
ASR	20.1	2.7	18.9	4.9	21.5	11.6	14.3	6.5

*1985–1989*								
No.	25	7	48	19	48	19	114	48
ASR	21.5	9.0	26.6	9.5	35.0	12.6	16.7	7.2

*1990–1994*								
No.	48	7	63	24	94	29	179	100
ASR	19.0	4.5	54.0	10.2	56.0	15.1	22.7	11.8

*1995–1998*								
No.	37	8	60	31	90	32	154	98
ASR	25.7	5.4	39.0	48.4	60.7	18.6	20.7	8.7

*1960–1998*								
No.	155	38	252	104	321	114	742	374
ASR	18.9	5.2	22.2	12.6	31.6	11.0	15.1	6.4

ASR: age-standardised rate.

). Overall, the Jews born in Africa demonstrated the highest incidence rates in men and the second for women, followed by high rates in Asian-born Jews. Few cases were registered for Jews originating from America.

Standardised ratio and relative risks (RRs) for specific representative countries of origin could be calculated only for the period between 1980 and 1994, with Jews born in Israel taken as reference (Table 4

**Table 4:** Relative risk of classic Kaposi's sarcoma by selected countries of origin and gender, 1980–1994

	**Men**		**Women**		**Relative risk**	
**Country**	**No. of cases**	**ASR**	**No. of cases**	**ASR**	**Men**	**Women**
Israel	89	2.02	17	0.54	1	1
FSU	75	1.48	53	0.95	0.7(0.94–2.341)	1.8(0.83–4.43)
Poland	147	2.20	57	0.77	1.1(0.96–3.16)	1.4(0.92–3.72)
Yemen	32	2.83	6	0.42	1.4(0.92–3.72)	0.8(0.95–2.55)
Iraq	70	3.62	35	1.67	1.8(0.83–4.43)	3.1(0.35–6.55)
Morocco	104	4.35	41	1.6	2.1(0.74–4.94)	3.0(0.39–6.39)
Romania	93	4.51	42	0.7	2.2(0.71–5.11)	1.3(0.93–3.53)

ASR: age-standardised rate; FSU: Former Soviet Union.

). Prior to and after 1980–1994, the data received from the Population Registry were gathered as groups of countries that could not be separated. In men, the highest incidence rate was observed among immigrants from Romania. In women, the highest incidence rate was observed among immigrants from Iraq.

## DISCUSSION

The present study, based on 2107 registered CKS cases among Jewish residents of Israel, has found an age-standardised incidence rate of CKS (±s.d.) of 20.7 (±9.3) per million among men and 7.5 (±3.4) per million in women. Similar rates of CKS were reported, for much narrower calendar periods, from Sardinia (Cottoni *et al*, 1996) and Sicily (Geddes *et al*, 1994). A rather constant gender ratio of 2.33 was found, which is in agreement with previous population-based surveys (Biggar *et al*., 1984; Dictor and Attewell, 1988; Grulich *et al*., 1992; Geddes *et al*, 1994).

Here we report higher rates compared with those previously reported for the Israeli Jewish population for the period 1961–1989 (16.9 per million in men and 6.3 per million in women), in which a total of 1098 cases of CKS were analysed (Iscovich *et al*, 1998). The higher rates described in our study can be explained by the addition of 1009 new CKS cases and by the vast changes in the Jewish population composition with the mass migration of approximately 1 000 000 new immigrants from the Former Soviet Union (FSU) during the late 1980s and the 1990s.

The present study demonstrates two steep rises in incidence. A similar pattern of increase in incidence rates during the early 1970s was described (Hjalgrim *et al*, 1996; Dictor and Attewell, 1988). Improved registration is likely to have played a certain role, but it cannot be the sole explanation for this finding. Differences in immigration patterns in Israel may provide another explanation. During the 1950s through the late 1960s, there were large immigration waves from Iraq and North African countries, and during the late 1960s and early 1970s there were large immigration waves from Eastern Europe.

The increase in incidence rates during the early 1990s can be explained by the fact that CKS is a slowly developing disease, and similar to other chronic diseases, migrants are more likely to be diagnosed with such disorders, as a result of entering a new health system. An additional explanation for this increase might be the aging of the population that arrived with the large immigration waves to Israel during the 1950s and the 1960s. Moreover, this could also be explained by the massive immigration wave from the FSU. Interestingly, the majority of the newly diagnosed cases of CKS between 1990 and 1998 were among Jews of East European origin, whereas CKS incidence rates between 1990 and 1998 were higher for Jews originating from Africa and Asia, despite the much lower number of newly diagnosed cases among them.

The significant difference in incidence rates by countries of origin suggests an important role for geographical differences in the prevalence of KSHV (Chatlynne and Ablashi, 1999; Schulz, 1999). Variation in KSHV seroprevalence among Israeli populations was found to be strongly associated with the country of birth (Davidovici *et al*, 2001). The highest rates of anti-KSHV antibodies were found in those born in North African countries, and the lowest rates were found in those born in Europe or North America (Davidovici *et al*, 2001). These findings correlate with the particularly high incidence of CKS observed in our study among North African Jewish immigrants in Israel.

In summary, our study supports an important role for the country of origin, which, together with environmental conditions, is likely to be of importance in influencing the exposure of individuals to KSHV, and may also be implicated in establishing the risk for the development of CKS. Furthermore, it could be possible that the seroprevalence rates of KSHV and CKS incidence rates among the various Israeli subpopulations reflect the situation in the countries of origin, and thus our population may serve as an ‘indicator population’. Still, since CKS that was originally described in Jews of eastern European descent is also highly prevalent in Jews from Asia and Africa, it may suggest that infection with KSHV had been relatively more prevalent among Jews before the Diaspora of Jewish people that started more than 2500 years ago.
